# Temporal patterns in Saturnidae (silk moth) and Sphingidae (hawk moth) assemblages in protected forests of central Uganda

**DOI:** 10.1002/ece3.1477

**Published:** 2015-03-25

**Authors:** Perpetra Akite, Richard J Telford, Paul Waring, Anne M Akol, Vigdis Vandvik

**Affiliations:** 1 Department of Biological Sciences, Makerere UniversityKampala, Uganda; 2 Department of Biology, University of BergenBergen, Norway; 3 Windall ViewWerrington, Peterborough, UK

**Keywords:** Compositional change, extinction debt, forest degradation, Lepidoptera, matrix intensification, resampling, species decline

## Abstract

Forest-dependent biodiversity is threatened throughout the tropics by habitat loss and land-use intensification of the matrix habitats. We resampled historic data on two moth families, known to play central roles in many ecosystem processes, to evaluate temporal changes in species richness and community structure in three protected forests in central Uganda in a rapidly changing matrix. Our results show some significant declines in the moth species richness and the relative abundance and richness of forest-dependent species over the last 20–40 years. The observed changes in species richness and composition among different forests, ecological types, and moth groups highlight the need to repeatedly monitor biodiversity even within protected and relatively intact forests.

## Introduction

For terrestrial ecosystems, the most important driver for biodiversity change in the last 50 years has been land cover change (e.g., Sala et al. 2000; Fahrig 2003; Foley et al. 2005; MEA 2005). In some regions, less than 10% of the original vegetation remains after clearance for agriculture and other purposes (Saunders et al. 1991). Tropical forests are among the habitats experiencing the highest loss rates. In addition to the area loss per se, deforestation has resulted in fragmentation of once-continuous forests; the resulting fragments are now surrounded by a matrix of other land-uses (Fahrig and Merriam 1994).

Species vulnerability to forest area loss and fragmentation is strongly affected by their ability to use these matrix landscapes (Gascon et al. 1999). Different species perceive the matrix differently: what is inhospitable to one may be habitable to another and what is a barrier to one may be easily traversed by another (Bowler and Benton 2005; Eycott et al. 2012). Consequently, ecological patterns and processes within patches may also be influenced by the nature of the surrounding matrix landscape (Ricketts 2001; Vandermeer and Carvajal 2001; Prugh et al. 2008) particularly in species exhibiting a metapopulation structure (Hanski 1998).

Species response to landscape changes is influenced by key ecological and life-history attributes such as longevity, reproductive rates, body size, trophic specialization, and dispersal ability. These traits directly determine changes in abundance and mediate extinction risk (Pimm et al. 1993). Identifying how species with different traits are differentially affected by landscape change allows insight into shifts in communities or guild composition beyond simple changes in species richness (Williams et al. 2010). One way to understand the mechanisms that determine the structure of communities is through the comparison of species diversity at different spatial and temporal scales in different ecological and biogeographical settings (Fukami and Wardle 2005). Temporal patterns of biodiversity have received much less attention than spatial ones (Magurran et al. 2010). Such comparisons are important in developing and planning conservation program (e.g., Kremen et al. 2007) and provide a vital tool for management of wildlife (Turner et al. 2003).

Insects make an enormous contribution to diversity and ecosystem functions (Lewinsohn et al. 2005) but knowledge of population changes in insects lags behind that of vertebrates and vascular plants (Thomas et al. 2004). Despite their importance for many critical ecological functions, unparalleled contribution to biodiversity, and their potential use in conservation planning, long-term ecological studies of invertebrates are extremely scarce (Kremen et al. 1993). For Lepidoptera, data on population size fluctuations and associated changes at community level are available for many temperate ecosystems but much less is known about their dynamics in the humid and seasonal tropics (e.g., Schulze and Fiedler 2003). Moths play a central role in many ecosystem processes as prey, herbivores, and pollinators (Janzen 1987; Barlow and Woiwod 1989). Saturnidae (Silk moths) and Sphingidae (Hawk moths) are two of the most species-rich families of moths in the tropics (Janzen 1984). They can be rapidly surveyed, identified and are relatively well documented, thus well placed to act as indicator groups. Members of the two families have differing life histories and feeding habits. Tropical Sphingidae are long-lived with a few exceptions, mate repeatedly, lay a few eggs per host plant and oviposit through adult life (e.g., Haber and Frankie 1989). They are accomplished fliers with migratory tendencies in some taxa. In contrast, Saturnidae have short non feeding flight periods, lasting less than 10 days, mate once and lay many eggs. They are often associated with undisturbed forest habitats. Caterpillars of Saturnidae often select older leaves and are usually found in crowns of adult trees or woody vines, while Sphingidae are less particular about plant age and commonly feed on young leaves (Janzen 1984). These factors make it likely that Sphingidae are typically better dispersers and less habitat specific than Saturnidae.

Most Ugandan forests and their matrix landscape have undergone considerable changes in recent decades (Obua et al. 2010). Many forests have been lost, but some are well protected and have experienced little structural and tree compositional change in recent decades (e.g., Bulafu et al. 2013). Our study aims to resample historical data on moth communities to give insights on the long-term ecological integrity of these protected forests in rapidly changing matrix landscapes. Based on the literature summarized above, we test the predictions that (1) moth species richness has declined over time; (2) forest-dependent species are more affected than the generalist species; and (3) Saturnidae will have declined more rapidly than Sphingidae.

## Materials and Methods

### Study areas

**Figure 1:**
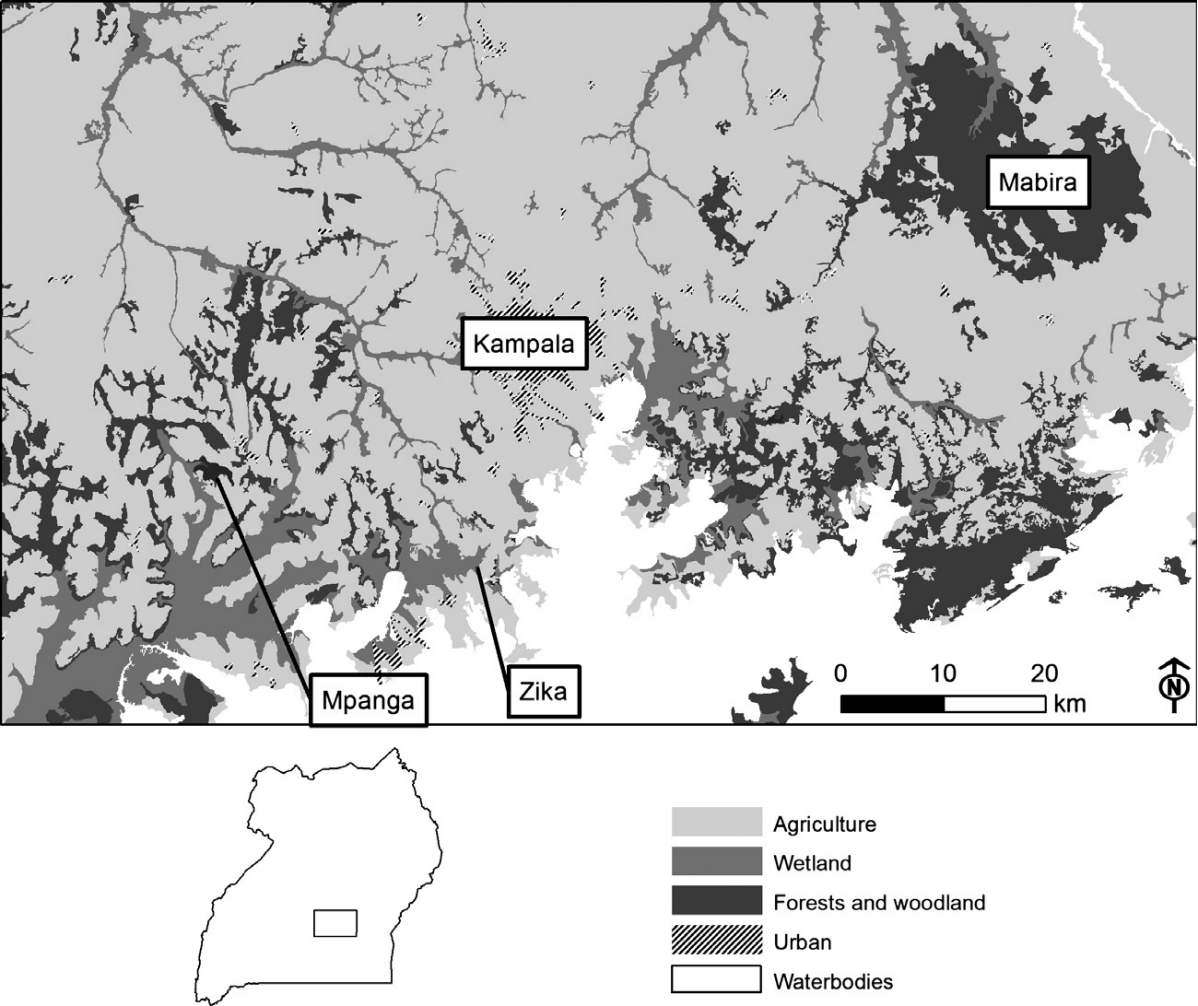
Location of the surveyed forests including major habitat types in the matrix.

The study was conducted in three protected forests in central Uganda: Zika, Mpanga, and Mabira (Fig.1). These forests are near Lake Victoria, in the wettest districts of central Uganda where mean annual rainfall ranges from 1200 to 1600 mm and mean annual temperature is 28°C (MWLE 2002). They lie within an elevational range of 1070 – 1340 m asl.

### Study sites

Zika (0°07′N, 32°31′E) covers an area of 0.13 km^2^. The forest is part of a narrow sinuous strip of lakeside forests skirting the extensive grass and papyrus swamps of Waiya Bay, a sheltered inlet of Lake Victoria. Buxton (1952) recognized three zones in the forest: a) permanent swamp forest dominated by *Mitragyna stipulosa*, *Erythrina excelsa,* and *Voacanga obtusa*; b) raised wet forest dominated by *Pseudospondias microcarpa*, *Parkia filicoidea,* and *Macaranga monandra*; c) raised seasonal forest dominated by *Lovoa brownie*, *Maesopsis eminii,* and *Piptadenia africana*. The forest has been under the jurisdiction of the Uganda Virus Research Institute since 1960.

Mpanga (0°15′N, 32°18′E) covers an area of 4.53 km^2^. It is a remnant tropical, medium altitude, moist evergreen, and swamp forest comprised of a) swamp – permanently flooded or water logged *Mitragyna*–*Phoenix* associations; b) the slopes with *Celtis*–*Aningeria* associations; and c) main forest dominated by *Pseudospondias microcarpa*, *Erythrina excelsa*, *Canarium schweinfurthii,* and *Entandrophragma angolense* (Buxton 1952). The forest was gazetted as a nature reserve in 1950 and is under the jurisdiction of the National Forestry Authority (NFA).

Mabira (0°24′–0°35′N, 32°52′–33°07′E) covers an area of 306 km^2^, the largest block of moist semi-deciduous forest remaining in central Uganda (Carswell 1986). The reserve is considered to be a secondary forest and Howard (1991) described four major types within this a) younger secondary forests dominated by colonizing *Maesopsis eminii*; b) valley bottom forest dominated by *Baikiaea insignis*; c) the *Celtis*–*Holoptelea* dominated forest; and d) mixed communities. As noted by Winterbottom and Eilu (2006) and from field observations, vast areas of the forest are now covered by the exotic species *Broussonetia papyrifera*. The forest is protected and managed as a Central Nature Reserve by the NFA.

Zika and Mpanga forests have remained relatively undisturbed and unchanged internally (Bulafu et al. 2013), but their surrounding matrix landscape is substantially altered; the majority of neighboring forest fragments in the greater Kampala–Entebbe landscape (Bulafu et al. 2013) and the Mpigi archipelago have either been cleared or greatly reduced in extent and/or quality. Parts of Mabira are recovering from the encroachments of the 1970s–1980s (MWLE 2008), and large sections of the nature reserve are relatively stable, albeit with minor disturbances from illegal logging, but the forest area has declined as edges have been lost to sugarcane, tea, cardamom or oil palm plantations, plus non-native monoculture forestry and small- to medium-scale agro-ecosystems (MWLE 2008).

To assess recent changes in the matrix, we calculated the percentage of the forest area in a 5-km buffer around each forest, and the loss of forest over the period 2000-2012 based on the remote sensing analysis of Hansen et al. (2013). We reclassify Hansen et al. (2013) so that cells with 60% forest cover or above are classified as forest to reduce misclassification of papyrus swamps near the forests. In 2000, there was 3% forest around Zika, 17% around Mpanga, and 5% around Mabira. Loss 2000–2012 around Zika was 2.3%, Mpanga 4.7%, and Mabira 1.7%.

### Field methods

The historic data have been sampled in two periods**.** In Zika forest, the Saturnidae were surveyed by Angus McCrae between February 1969 and April 1971 (unpublished) and by the Uganda Forest Department (FD) between March 1993 and January 1995 (Howard and Davenport 1996). The FD surveys included the Sphingidae. In Mpanga, the FD carried out moth surveys between September 1993 and February 1995 and in Mabira, the surveys were carried out between October 1992 and February 1995 (Howard and Davenport 1996).

McCrae sampled the moths using a single Robinson trap powered by a portable generator and operated from dusk to dawn. Short-term trapping was done in the period February to March 1969, followed by an intensive study from April 1969 to April 1971 (unpublished). The moths were identified by McCrae, and all voucher specimens from this study are deposited at the Hope Museum in Oxford.

The 1990s data come from short-term surveys and a more intensive sampling program run by the FD. The moths were sampled using a 125-watt choked mercury vapor lamp mounted in a Skinner box trap. The trap was powered by a portable generator and operated from dusk to dawn. The FD surveys were conducted to be as similar as possible to the earlier study by McCrae. All moths were identified by Peter Howard in consultation with McCrae, and voucher specimens are deposited at the Makerere University Zoology Museum. Howard and Davenport (1996) give detailed findings of sampling in each forest, including dates, captures per night, and total trap nights.

The resampling data were collected for the two moth families in the three forests from September 2010 through March 2011. Traps were placed away from the edge by at least (≥100 m) except in the much smaller Zika forest where traps were placed in the center of the forest. We surveyed the same localities as Howard and Davenport (1996) and trap locations remained the same throughout the entire survey. In Zika, sampling was carried out in the periods 3–11 January then 13–23 February 2011; in Mpanga, sampling was carried out in the periods 15–29 December 2010 then 13–23 February 2011; and in Mabira, sampling was carried out in the period 9 September – 10 December 2010. Sampling was carried out using a portable light-trap consisting of a 15-watt actinic tube (Sylvania blacklight F 15 w/BLB–TB) run on a portable car battery (32 amps) and a net and was run from dusk to dawn. Actinic traps are known to be highly effective and minimize cross attraction of moths between sampled habitats (Muirhead-Thomson 1991; Schulze and Fiedler 2003). Each morning, special attention was given to searching the area around the trap for any moths that had been attracted to the light, but had not entered the trap; individuals of the target families were hand-collected.

Specimens of the two families were first photographed at the trap, and then, representative samples collected and dried in envelopes. Voucher specimens were examined and identified (where possible) with reference to Pinhey (1972) and D'Abrera (1986), and the reference collections from the FD surveys. Clarification was sought for some species by checking the extensive collections of A. McCrae at The Hope Museum (Oxford, UK) and the collections at The Natural History Museum London (NHM). Some of the Sphingidae were identified by Ian Kitching.

### Data combination and species classification

The sampling effort differed between the sampling periods. The 1970s and 1990s species lists are extensive – a large fraction of the species that occurred in (and possibly around) these forests were probably recorded. To achieve meaningful comparisons, subsets of data from the 1970s and 1990s were extracted which covered only the same portion of the year as the 2010s surveys (Table1). These are referred to as the 1970, 1990, and 2010 data hereafter.

**Table 1:** Moth total abundance, mean number of individuals per trap night, number of species, and the exponent of the Shannon in the three forests

Moth family	Forest sampled	Sample period	Year	Total trap nights	Number of moths	Number of species	Mean number of individuals per night	expH'
Saturnidae	Zika	MaCrae's subset	1969–1971	161	1765	54	11.0	25.0
		Forest Dept subset	1993–1995	82	474	33	5.8	17.4
		Resample	2011	12	131	20	10.9	15.2
	Mpanga	Forest Dept subset	1993–1995	27	161	32	6.0	24.7
		Resample	2010–2011	18	151	26	8.4	23.0
	Mabira	Forest Dept subset	1992–1995	98	528	40	5.4	24.0
		Resample	2010	42	427	35	10.2	28.1
Sphingidae	Zika	Forest Dept subset	1993–1995	82	1143	46	13.9	23.2
		Resample	2011	12	196	34	16.3	29.3
	Mpanga	Forest Dept subset	1993–1995	27	500	46	18.5	20.7
		Resample	2010–2011	18	321	29	17.8	23.4
	Mabira	Forest Dept subset	1992–1995	98	867	39	8.8	16.5
		Resample	2010	42	524	29	12.5	17.6

Howard and Davenport (1996) describe the ecological habitat preferences of all species they observed based on prior knowledge of their ecology (Carcasson 1976). Habitat preferences include: F) forest-dependent species restricted to closed-canopy forest habitats; f.) forest nondependent species not infrequently recorded in closed-canopy forest, but also encountered in a variety of forest edge, degraded forest, and woodland habitats; G) nonforest species characteristic of open habitats such as grassland, open savannah, and arid habitats; and W) widespread species, generalist that occur in a variety of forest and nonforest habitats.

### Data analysis

Trap data were pooled for each of the two moth families in each forest per sampling period. We calculated the exponent of the bias-corrected Shannon index (Chao and Shen 2003) for each sample period, called the “effective number of species” of the community (Hill 1973). This index converges rapidly with little bias even for small samples (Magurran 2004).

The observed number of species is a misleading indication of species richness because of the difficulty of obtaining a complete inventory of species-rich communities (Price et al. 1995). Individual-based rarefaction curves were therefore used to evaluate the effectiveness of sampling and for comparison of species accumulation curves following Gotelli and Colwell (2001). For the statistical comparison of the accumulation curves, we calculated the rarefied number of species and the 95% confidence interval using bootstrap resampling with replacement using EstimateS Version 9.1.0 (Colwell 2006).

The proportion of the total moth fauna belonging to each ecological habitat preference types was calculated based on abundance and presence/absence data. We performed a chi-square test for homogeneity to check whether the observed differences in richness and abundances of the different ecotypes over time within the two moth families are significant. Data were analyzed using the statistical program R (v. 2.13.1, R Core Team 2013).

## Results

The combined dataset consists of 3687 individuals (54 species) of Saturnidae and 1041 individuals (49 species) of Sphingidae across the three forests. The number of moths differed with effort between sampling periods, but the mean numbers of individuals per trap night are generally comparable across the sampling periods (Table1).

**Figure 2:**
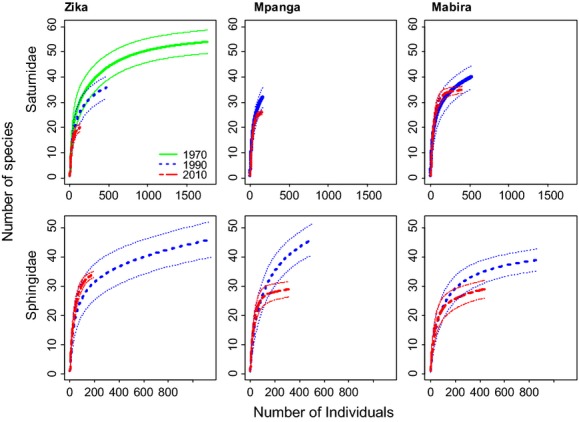
Individual-based rarefaction curves for the moths (± CI 95%; Green = 1970, Blue = 1990, Red = 2010), for three different forest reserves in central Uganda.

There was no overall pattern in the effective number of species over time (Table1). Visual assessment of the rarefaction curves reveals that many of the curves do not approach asymptotes (Fig.2). Generally, the curves in the 2010 resampling were lower than that in the earlier periods (Fig.2). Comparing species accumulation curves (CI: 95%) at the lowest abundance value (2010 resample period) show clear decreases for the Sphingidae in Mpanga and Mabira, less conclusive patterns for Saturnidae in Zika and Mpanga, and no support for change for Saturnidae in Mabira and Sphingidae in Zika (Fig.2).

**Figure 3:**
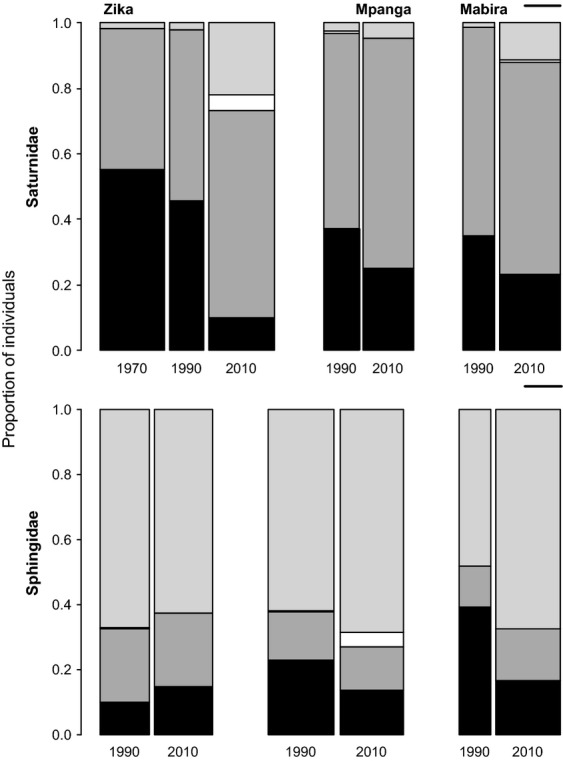
Proportional changes in composition based on individual abundances of each ecotype over time. Black = forest-dependent species; dark gray = forest nondependent species; white = open habitat species, and light gray = widespread species. The width of the bars is proportional to the total number of individuals. The small black line on the right represents eight individuals per trap night.

The proportions of individuals belonging to forest-dependent species are greatly reduced in the 2010 sampling period compared with the earlier sampling periods (Fig.3). Proportion varied significantly with sampling period among the Saturnidae in Zika (*χ*^2^ = 297.81, df = 6, *P *< 0.0001, Fig.3) and Mpanga (*χ*^2^ = 56.37, df = 3, *P *< 0.0001, Fig.3), and also among the Sphingidae in Mpanga (*χ*^2^ = 26.55, df = 3, *P* < 0.0001, Fig.3) and Mabira (*χ*^2^ = 100.73, df = 2, *P *< 0.0001, Fig.3). Saturnidae in Mabira show a marginally significant decline (*χ*^2^ = 6.94, df = 3, *P* = 0.07, Fig.3), and there was no statistical difference among the Sphingidae in Zika (*χ*^2^ = 4.50, df = 3, *P* = 0.212, Fig.3).

**Figure 4:**
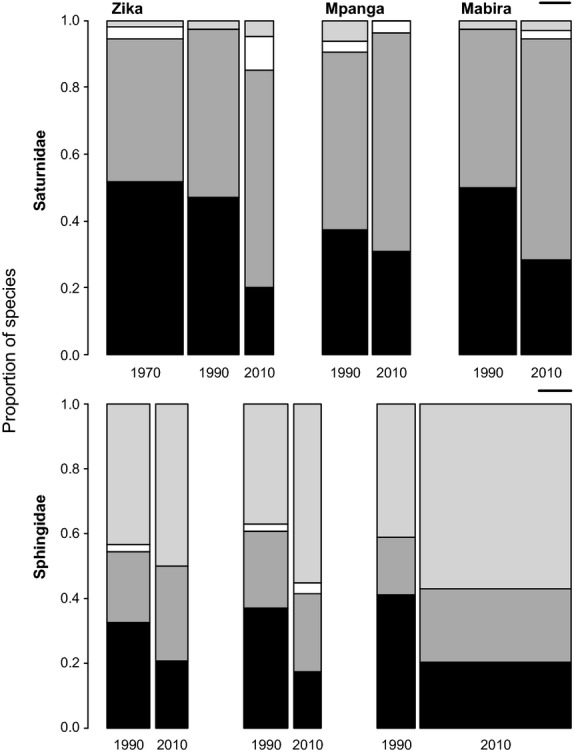
Proportional changes in composition based on total number of species of each ecotype over time. See Fig.3 for legend. The width of the bars is proportional to the total number of species; the black lines on the right represent 12 species.

The same pattern is reflected in the species richness, with an increase in the proportions of forest edge and widespread species and a decline in the forest-dependent species (Fig.4). This is only statistically significant for Sphingidae in Mabira (*χ*^2^ = 7.35, df = 2, *P* = 0.025, Fig.4), whereas there was no statistical difference for Saturnidae (Zika: *χ*^2^ = 8.78, df = 6, *P* = 0.175; Mpanga: *χ*^2^ = 4.40, df = 3, *P* = 0.221; Mabira: *χ*^2^ = 2.20, df = 3, *P* = 0.531, Fig.4) and for Sphingidae in Zika (*χ*^2^ = 2.41, df = 3, *P* = 0.492, Fig.4) and Mpanga (*χ*^2^ = 3.81, df = 3, *P* = 0.283, Fig.4).

There was turnover in species over the years (Table2). Nine species of Saturnidae from the 1970s in Zika are absent from the 1990s resampling with one rediscovered in the 2010 sampling period. *Orthogonioptilum luminosum*, relatively abundant in Zika forest in the 1970s, has not been recorded in the subsequent 1990 and 2010 resample periods. Two species, *Temnora hollandi* and *Imbrasia oyemensis,* known only from the Kampala–Entebbe area in the East African parts of their ranges and only recorded in Zika in the 1970s (Angus McCrae, personal notes) were absent from the 1990 and 2010 resampling.

**Table 2:** Number of Saturnidae and Sphingidae species in the different sampling periods

Categories	Zika		Mpanga		Mabira	
	Saturnidae	Sphingidae	Saturnidae	Sphingidae	Saturnidae	Sphingidae
1970 only	19					
1970 + 1990	15					
All three samples	18					
1970 + 2010	2					
1990 only	0	17	10	22	12	16
1990 + 2010	0	29	20	24	28	23
2010 only	0	5	6	5	7	7

## Discussion

Despite only minor changes in forest structure and vegetation of our forests in recent decades (Obua et al. 2010; Bulafu et al. 2013), we observe large changes in moth communities. Although we do not have good estimates of the total moth richness, rarefaction curves suggest declining diversity. The most striking pattern is the change in the ecotype composition of the forests with consistent decline in the relative abundance of forest-dependent species and an associated increase in widespread species. In line with our predictions, Saturnidae were more affected. These declines in moths reported here are in line with patterns of moth declines recorded elsewhere around the world (e.g., Great Britain, Conrad et al. 2004; Finland, Huldén et al. 2000). Our results give some insights into the ecological processes operating in these forests and the surrounding landscapes.

Although sampling protocols and sampling efforts varied between the sampling periods, the number of individuals per trap night was comparable across the sample periods, suggesting that different trap types have approximately similar efficiencies. Summerville and Crist (2005) found that differences among trap types contributed less than 10% to differences in richness. A similar study on moths in a lowland dipterocarp forest in Peninsular Malaysia using two different trap types (Intachat and Woiwod 1999) found no significant differences between overall diversity for geometroidea between the trap types and that total catches for nongeometroidea were remarkably similar. The reduced cross-habit attraction of actinic traps (Muirhead-Thomson 1991; Schulze and Fiedler 2003) will bias our results, but the direction of bias is opposite to the patterns we observe. Regarding sampling effort, a comparison between long-term sampling and short-term but intensive sampling yielded a 76% overlap in species recorded (Landau et al. 1999). Similarly, Summerville and Crist (2005) reported that increased sampling effort only reduced the proportion of singletons and unique species and this peaked off after 10 trap nights. Therefore, we believe that the observed changes in rarefied species richness and species turnover among the Saturnidae and Sphingidae within these protected forests are real.

The weak patterns in overall species richness and diversity reflect the replacement of forest-dependent species with widespread species. Thus, the dramatic decline of forest-dependent species relative abundance and richness is masked by the rise of the widespread and generalist taxa. This highlights the limited utility of diversity metrics for conservation; indeed, they can be misleading.

Changes in moth diversity and abundance have often been correlated with or assumed to be caused by environmental changes within the study sites (Summerville et al. 2004). Despite past encroachment, these forests have either remained relatively stable in size and structure (Zika and Mpanga; Bulafu et al. 2013) or have been recovering from disturbance (Mabira) over the resampling period (Winterbottom and Eilu 2006; Obua et al. 2010). It is therefore unlikely that the observed declines in forest specialist species are only driven by environmental change within the forests.

Alternative explanations for the observed patterns in moth community within our study forests include the effect of increased isolation due to matrix transformation, extinction debt, and climate change. Habitat isolation in both space and time disrupts species distribution patterns, consequently affecting metapopulation dynamics of patch-dwelling populations. This makes matrix habitats strong determinants of fragmentation effects within remnants through regulating dispersal, dispersal-related mortality, and mediating edge-related microclimatic gradients (Ewers and Didham 2006). Such consequences of isolation may be heightened in Zika forest where Bulafu et al. (2013) reported a 50% loss of all its neighboring forests over the last 20 years despite the forest itself remaining stable with very low levels of disturbance. This is supported by the remote sensing forest loss analysis where over 60% of the forest area in a 5-km buffer around Zika has been lost, the highest proportion of any of our forests (Hansen et al. 2013).

Rapid expansion and intensification of agriculture, coupled with loss and deterioration of suitable habitats, have been implicated in the decline of moths elsewhere (e.g., Conrad et al. 2004; Fox 2012) and several other insect groups (e.g., dung beetles, Nichols et al. 2007; butterflies, Ekroos et al. 2010). We found significant declines in the moths' richness and relative abundance of forest-dependent species especially in the poorly dispersed Saturnidae. In central Uganda, there has been massive intensification in the use of the matrix surrounding protected forests; previously, forested areas have been replaced by exotic plantations (e.g., oil palm, cardamom, or eucalyptus), agro-ecosystems (e.g., shade coffee and home gardens), or cleared for settlements and other human developments (Obua et al. 2010). Matrix intensification can lead to a breakdown in metapopulation dynamics, making land-use-driven environmental changes outside reserves just as important as those within reserves in determining the fate of regional-scale biodiversity (e.g., Hanski 1998; Perfecto and Vandermeer 2002). For example, a study on birds in the farmed landscapes of Central and southwestern parts of Uganda reported declines with increased land-use intensification especially among forest specialist species (Bolwig et al. 2006).

In tropical forests where historical forest area loss and landscape change are high, extinction debt might drive species loss long after forest reserve boundaries have stabilized (e.g., Kuussaari et al. 2009). The magnitude of extinction debt that can be expected is largely dependent on spatiotemporal configuration of habitat patches, the time since the habitat was altered and the nature of the alteration (Kuussaari et al. 2009) but also on the life-history traits of the assemblages. We expect that extinction debt would be repaid fastest in the small Zika forest, with its consequently small populations, leading to high rates of species loss.

Rainfall patterns in central Uganda have changed in recent decades, resulting in either less precipitation or alteration in timing of the rainy season (e.g., MWLE 2002; Williams and Funk 2011; Michaelsen and Marshall 2012). This would affect adult moth emergence, often triggered by rainfall signaling larval food availability. Pronounced fluctuations in the abundance of individual species or entire guilds of moths over seasons are reported to be frequent (e.g., Fiedler and Schulze 2004). Climate effects have been reported in Britain and northwestern Europe, where substantial decreases in the overall abundance of macro-moths and populations of many widespread species have been attributed to habitat loss in combination with climate change (Conrad et al. 2006; Fox 2012).

### Compositional change in ecological types

In general, habitat specialists are more susceptible to habitat loss and degradation than generalists (e.g., Öckinger et al. 2010). In the earlier sampling periods, forest-dependent species especially those associated with woody plants were prevalent and abundant (McCrae unpublished, Howard and Davenport 1996). Our 2010 data are characterized by species that have wider ranges and do not need good quality forests to survive.

The observed declines in forest-dependent species vs. increases in forest edge and widespread species within our study forests are indicative of the ability of nonspecialist species to utilize a much broader range of habitats across the landscape compared to the specialist species that require intact and less disturbed habitats for their survival. In a connected landscape, forest-dependent species may benefit from reinforcement between fragments as a result of metapopulation dynamics rescuing species from imminent extinction (Nee and May 1992; Hanski 1998). The severity of deforestation in our study area will clearly reduce the opportunities for forest-dependent species to cope with the changes in their environment.

Kitching et al. (2000) and Usher and Keiller (1998) both note that forest specialist moths tend to be monophagous and feed on woody plants, trees, and vines, whereas moths that favor disturbed sites are often polyphagous, feeding on herbaceous and weedy food plants. This could potentially account for some of the variability in our data. Individual species' responses can thus be interpreted through their guild membership – as forests are lost or altered, monophagous (forest specialist) species are more likely to decline or go locally extinct than those that are polyphagous (i.e., generalist, Holloway and Hebert 1979). Several previously common forest-dependent species were absent from subsequent resampling periods (e.g., *Imbrasia anthina*), while forest edge and widespread species (e.g., *Cirina forda* and *Imbrasia anna*) became more common. The Parasol tree, (*Polyscias fulva*) which is a larval food plant for *Imbrasia anthina*, is now scarcely found in its natural habitat within our study forests as result of unsustainable harvesting for making drums (Omeja et al. 2005; Were 2010), and this species of moth was missing in the 2010 resample period. *Imbrasia anna* which feeds on several members of the Arecaceae family had larger populations in our dataset and has a wide geographic range compared to *Imbrasia oyemensis* which is a forest-dependent species that only feeds on *Entandrophragma angolense* – an IUCN red listed tree species. However, a shortage of trait data for most species of tropical Africa, especially larval host plants, hinders our inferences in this regard.

### Saturnidae and Sphingidae

We found steeper declines among the Saturnidae than the Sphingidae. This is in accordance with our expectations based on the lifestyle and dispersal ability of the two families. Saturnidae caterpillars tend to feed on older leaves and are often found in the crowns of trees, whereas the Sphingidae tend not to be particular about plant age and commonly feed on younger leaves (Holloway and Hebert 1979; Bernays and Janzen 1988). This predisposes Saturnidae to greater impacts from habitat disturbance and especially when mature trees and woody vines are continuously taken out of their ecosystems (Basset 1992; Kitching et al. 2000).

## Conclusions

Our results show some significant change in the moth communities in the target forests in the last 20–40 years. This highlights the need to repeatedly monitor biodiversity even within protected and relatively intact forests. Our findings together with similar patterns reported for trees (Bulafu et al. 2013) in similar habitat settings indicate a worrying reduction in the capacity of protected forests in central Uganda to maintain biodiversity. Matrix intensification around our forests appears to have reduced the capacity of the landscape to buffer and support populations in protected forests. Removal of any functional groups will alter the ecological integrity of these forests. In our case, if an important fraction or entire guilds of species are lost (e.g., forest-dependent species as our data suggest), detrimental effects on ecological services mediated by these moths could become apparent. Protected forests are linked ecologically to their surrounding habitats, and failure to stem broad-scale loss and degradation of such habitats could sharply increase the likelihood of serious biodiversity declines.
